# Nutritional Status Predicts 10-Year Mortality in Patients with End-Stage Renal Disease on Hemodialysis

**DOI:** 10.3390/nu9040399

**Published:** 2017-04-18

**Authors:** Shin Sook Kang, Jai Won Chang, Yongsoon Park

**Affiliations:** 1 Department of Dietetics and Nutrition Service, Asan Medical Center, 88, Olympic-ro 43-gil, Songpa-gu, Seoul 05505, Korea; sskang@amc.seoul.kr; 2 Division of Nephrology, Department of Internal Medicine, Asan Medical Center, University of Ulsan College of Medicine, 88, Olympic-ro 43-gil, Songpa-gu, Seoul 05505, Korea; jwchang@amc.seoul.kr; 3 Department of Food and Nutrition, Hanyang University, 222 Wangsimni-ro, Seongdong-gu, Seoul 04763, Korea

**Keywords:** protein energy wasting, mortality, hemodialysis, nutritional parameters

## Abstract

Protein-energy wasting (PEW) is associated with mortality in patients with end-stage renal disease (ESRD) on maintenance hemodialysis. The correct diagnosis of PEW is extremely important in order to predict clinical outcomes. However, it is unclear which parameters should be used to diagnose PEW. Therefore, this retrospective observational study investigated the relationship between mortality and nutritional parameters in ESRD patients on maintenance hemodialysis. A total of 144 patients were enrolled. Nutritional parameters, including body mass index, serum albumin, dietary intake, normalized protein catabolic rate (nPCR), and malnutrition inflammation score (MIS), were measured at baseline. Fifty-three patients died during the study. Survivors had significantly higher nPCR (1.10 ± 0.24 g/kg/day vs. 1.01 ± 0.21 g/kg/day; *p* = 0.048), energy intake (26.7 ± 5.8 kcal/kg vs. 24.3 ± 4.2 kcal/kg; *p* = 0.009) and protein intake (0.91 ± 0.21 g/kg vs. 0.82 ± 0.24 g/kg; *p* = 0.020), and lower MIS (5.2 ± 2.3 vs. 6.1 ± 2.1, *p* = 0.039). In multivariable analysis, energy intake <25 kcal/kg (HR 1.860, 95% CI 1.018–3.399; *p* = 0.044) and MIS > 5 (HR 2.146, 95% CI 1.173–3.928; *p* = 0.013) were independent variables associated with all-cause mortality. These results suggest that higher MIS and lower energy intake are harmful to ESRD patients on maintenance hemodialysis. Optimal energy intake could reduce mortality in these patients.

## 1. Introduction

Protein-energy wasting (PEW) is associated with an increased morbidity and mortality in patients undergoing maintenance hemodialysis for end stage renal disease (ESRD) [1]. PEW results from reduced dietary intake, inflammation, resistance to anabolic hormones, loss of nutrients during dialysis, and the breakdown of muscle protein induced by metabolic acidosis and comorbid conditions due to uremia [2]. In particular, a number of comorbid diseases and conditions are not only predictors of outcome [3], but also contributors to PEW in ESRD patients [4].

The prevalence of PEW among maintenance hemodialysis patients varies from 30% to 75% [5,6,7]. The diagnosis of PEW is important to predict outcomes [8]. The practice guidelines and criteria for evaluating the nutritional status in ESRD patients recommend the coordinated use of biochemical measures, body mass, muscle mass, dietary intake, and an integrative nutritional scoring [9,10]. However, it is unclear which method is better to evaluate PEW in ESRD patients on maintenance hemodialysis [11,12].

Inadequate dietary intake, which is considered the single most important cause of malnutrition in dialysis patients, is largely attributed to uremia secondary to inadequate dialysis [13]. Low protein and energy intake is frequently observed in maintenance hemodialysis patients [14,15,16]. Several studies reported that supplementation of protein and energy improved outcome, such as reduction of mortality and hospitalization in malnourished patients with maintenance hemodialysis [17,18,19]. Prospective studies also showed that the serum level of albumin was increased in malnourished patients on hemodialysis who received oral nutritional supplement [20,21]. Therefore, inadequate intake of protein and energy could be a major determinant in the development of malnutrition [14,16,22,23]. However, the impact of dietary intake or comorbidities on mortality has not been evaluated in ESRD patients on maintenance hemodialysis [12,24,25,26].

Antunes et al. [27] previously reported that protein intake <1.2 g/kg was predictive of 38-month mortality among dialysis patients. Araujo et al. [28] found that lower energy intake at the start of hemodialysis was a risk factor for 10-year mortality in ESRD patients. However, neither study included any data regarding the presence of comorbidities that affected the nutritional condition of these hemodialysis patients [28,29]. Therefore, the purpose of this study was to investigate which parameters were significant to predict 10-year all-cause mortality in ESRD patients on maintenance hemodialysis among nutritional markers, comorbidities, and anthropometric and biochemical parameters.

## 2. Materials and Methods

### 2.1. Patient Population

A total of 168 ESRD patients (age ≥ 20 years) on maintenance hemodialysis for at least three months were selected from the dialysis unit at Asan Medical Center (Seoul, Korea) during April 2006. Patients were undergoing 3.5–4.5 h dialysis sessions three times per week. The blood flow rate ranged from 200 to 350 mL/min, and a bicarbonate buffer was used. Patients were excluded if ascites (*n* = 6), malignancy (*n* = 3), severe infection (*n* = 6) or missing data regarding nutritional factors (*n* = 9) were identified during the assessment. Thus, 144 patients were enrolled in the study. The Charlson comorbidity index (CCI) was used to assess the presence of comorbidities [30].

The medical records of each maintenance hemodialysis patient were thoroughly reviewed. Any data pertaining to underlying kidney disease, cardiovascular history, or other comorbid conditions were extracted. This study was approved by the Asan Medical Center Institutional Review Board (2013-0074). The need for written informed consent was waived.

### 2.2. Follow-Up and End Points

The follow-up period began on the date of enrollment and finished at time of death from any cause or 30 April 2016, whichever came first. The survival time was measured in months from the beginning of the study until death or lost to follow-up. Mortality data were obtained from our medical records at Asan Medical Center or the Korea National Statistical Office. Ten patients were lost to follow-up because of cessation of peritoneal dialysis (*n* = 1), renal transplantation (*n* = 7) and uncompleted data (*n* = 2).

### 2.3. Anthropometric Measures

Anthropometric measurements were obtained within 5–20 min of the termination of hemodialysis. Body mass index (BMI; kg/m^2^) was calculated using dry weight. Body composition was determined by bioimpedance analysis (BIA; Inbody 720^®^, Biospace, Seoul, South Korea) approximately 30 min post-dialysis. Anthropometric measurements, muscle mass (MM), total body fat (TBF), and percent body fat (PBF) were measured using BIA. The total body fat index (TBFI) and muscle mass index (MMI) were calculated and expressed as kg/m^2^. Biceps-skinfold and triceps-skinfold thicknesses (TSF) were measured using a conventional skinfold caliper with standard techniques, as previously described [31]. The mid arm circumference (MAC) was measured using plastic tape, and the mid arm muscle circumference (MAMC) was calculated with the following equation [32]:

MAMC (cm) = MAC (cm) − (π × TSF).

### 2.4. Laboratory Evaluation

Blood samples were collected prior to the midweek hemodialysis session and included complete blood count, creatinine, urea, albumin, pre-albumin, calcium, phosphorous, total iron binding capacity (TIBC) and C-reactive protein. The normalized protein catabolic rate (nPCR) was calculated using single-pool urea kinetic modeling from two urea blood samples [33]. The Kt/V (urea clearance over time) was obtained using the second-generation Daugirdas formula [34]:

Kt/V = −ln(R − 0.008 × t) + (4 − 3.5R) × UF/W,

where R = post-dialysis/pre-dialysis blood urea nitrogen, t = dialysis hours, UF = pre-post-dialysis weight change, and W = post-dialysis weight.

### 2.5. Malnutrition Inflammation Score (MIS)

The MIS has four sections (medical history, physical examination, BMI and laboratory values) and ten components. Each component has 4 levels of severity from 0 (normal) to 3 (severely abnormal). Thus, the sum of all ten MIS components can range from 0 (normal) to 30 (severely malnourished); higher score reflects more severe degree of malnutrition and inflammation [35]. Five medical history-based components include dry weight change for the past 6 months, dietary intake, gastrointestinal symptoms, functional capacity and comorbid conditions. Two physical examination components consist of decreased fat stores or loss of subcutaneous fat and signs of muscle wasting. For each of these two components, a score of 0 through 3, represents normal to severe changes according to conventional subjective global assessment guidelines [36]. MIS-unique sections include BMI, serum level of albumin and TIBC, which are also scored from 0 through 3, respectively.

### 2.6. Dietary Assessment

The dietary intakes were assessed using a 24-h dietary recall on non-dialysis days [37]. Dietary intake of protein and energy was assessed from personal interview by one experienced renal dietitian. Energy and protein intakes were evaluated using a Computer-Aided Nutritional Analysis (CAN) software version 3.0 (Korean Nutrition Society, Seoul, South Korea, 2006). Dietary energy and protein intakes were normalized for adjusted body weight.

### 2.7. Statistical Methods

Continuous variables are presented as mean ± standard deviation (SD). Categorical variables are presented as frequency, percentage or ratio. Student’s *t*-test was used to detect significant differences between continuous variables between two groups. The Chi-square test was used for non-parametric variables, including sex and cause of ESRD.

We analyzed the nutritional cutoff points suggested by the International Society Renal Nutrition Metabolism, BMI < 23 kg/m^2^, energy intake <25 kcal/kg, protein intake <0.8 g/kg, albumin <3.8 g/dL [10] and nPCR 1.0–1.4 g/kg/day vs. <1.0 g/kg/d or >1.4 g/kg/day [38]. A cutoff point of MIS > 5 was chosen based on median values obtained in our studied population.

The Kaplan–Meier method was used to calculate cumulative survival probabilities, and the difference between survival curves was assessed by the log rank test. Cox proportional hazard analysis was used to evaluate independent nutritional predictors of survival. Independent variables for survival were analyzed between two groups stratified by presence or absence of malnutrition. The variables with *p*-value > 0.10 in univariate analysis were included as independent variables for multivariable analysis. In addition, the non-nutritional multivariable factors such as age, sex, Kt/V, diabetic mellitus and CCI were adjusted in the multivariable Cox proportional hazard model. The adjusted hazard ratios (HR) with 95% confidence intervals (CI) were reported. The Statistical Package for Social Sciences (SPSS 21.0 for Windows; SPSS, Chicago, IL, USA) was used in all statistical analyses. *p*-values ≤ 0.05 were considered statistically significant.

## 3. Results

### 3.1. Comparisons between Survivor and Non-Survivor Patients

Out of 144 patients, 53 patients (27%) died during the 10-year follow-up. The major cause of death was cardiovascular disease (Table 1). The major cause of ESRD was diabetic mellitus in both the survivor and non-survivor groups. The survivor group was younger, had a higher proportion of female patients, higher Kt/V, and lower CCI compared to the non-survivor group.

The MAMC was significantly higher in the only male survivor sub-group than it was in the other sub-groups. There were no significant differences in other anthropometric measurements or body composition between the two sub-groups classified by sex (Table 2).

With regard to the MIS and laboratory data, the survivor group had a significantly lower MIS score, higher nPCR, and higher calcium levels than the non-survivor group (Table 3). However, the other variables were not significantly different between the two groups.

The survivor group had significantly higher energy intake, higher protein intake, and higher ratio of protein intake over protein requirement than the non-survivor group (Table 4). However, there were no significant differences between the two groups with regard to the proportions of carbohydrate, protein, and fat.

### 3.2. Cox Proportional Hazards Analysis of Mortality

In univariate Cox regression analysis, all-cause mortality of ESRD patients on maintenance hemodialysis was positively associated with age, male sex, diabetes mellitus, Charlson’s comorbidity index, Kt/V, MIS > 5, energy intake <25 kcal/kg and protein intake <0.8 g/kg (Table 5). In multivariable Cox proportional hazard analysis, after adjusting for age, male sex, Kt/V, diabetic mellitus and CCI, all-cause mortality was positively associated with MIS > 5 and energy intake <25 kcal/kg body weight (Table 6). All-cause mortality was not associated with protein intake <0.8 g/kg body weight.

### 3.3. Kaplan–Meier Survival Curves Showing 120-Month Survival According to Nutritional Markers

**Figure 1:**
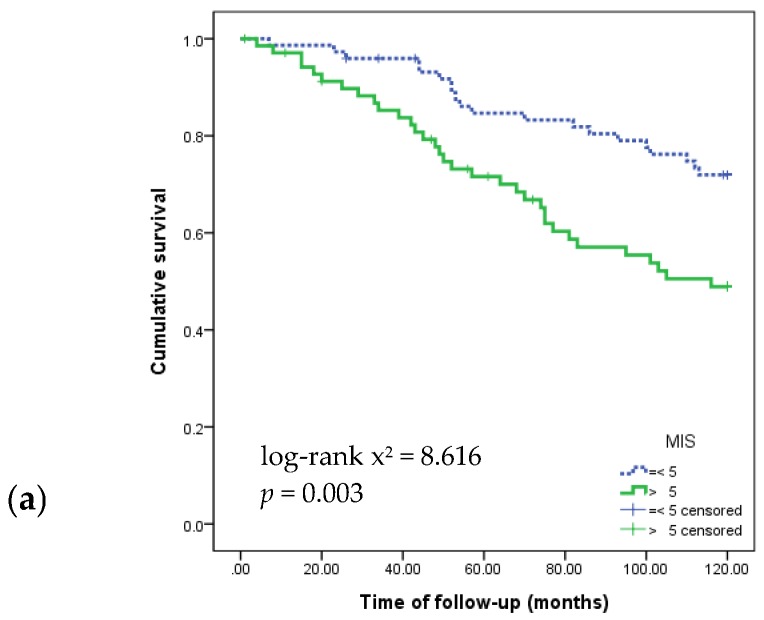

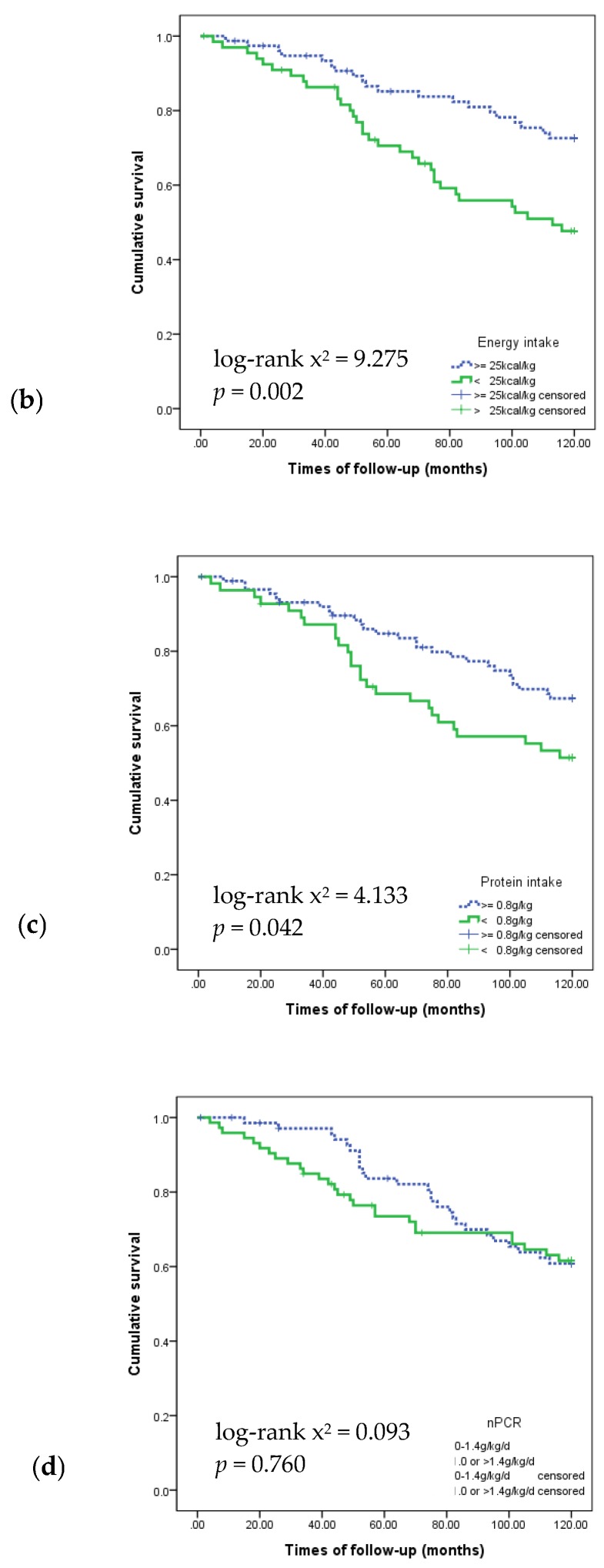
Kaplan–Meier survival curves showing 120-month survival according to nutritional markers: (**a**) Malnutrition Inflammation Score; (**b**) energy intake; (**c**) protein intake; and (**d**) nPCR.

Figure 1 shows the Kaplan–Meier survival curves for 120 months according to MIS, energy intake, and protein intake. The survival rate was significantly lower in patients with MIS > 5, protein intake <0.8g/kg and energy intake <25 kcal/kg.

## 4. Discussion

This was the first 10-year longitudinal retrospective study that evaluated the PEW parameters of ESRD patients on maintenance hemodialysis. Our data revealed that higher MIS (>5) and lower energy intake (<25 kcal/kg) could be associated with all-cause mortality in patients on maintenance hemodialysis.

MIS has been shown to be the best predictor of mortality in hemodialysis patients [12,39]. One study found that an MIS score >5 significantly increased the risk of one-year mortality in Asian patients with ESRD on maintenance hemodialysis [40]. In our study, the hazard ratio of death in patients with an MIS > 5 was 2.17 fold higher than those with MIS ≤ 5. Unfortunately, it is difficult to decrease the MIS, especially given that it has 10 components. On the other hand, lower energy intake increased the adjusted risk of death by 1.83 fold in the present study, which could be relatively easy to modify by increasing the amount of energy intake in ESRD patients on maintenance hemodialysis. This finding regarding energy intake is consistent with those of Araujo et al. [28], who found that energy intake (HR 0.96, 95% CI 0.92–0.99; *p* = 0.03) was an independent predictor of 10-year mortality at the beginning of hemodialysis in ESRD patients. Insufficient energy intake, even with adequate protein intake, could result in a negative nitrogen balance in hemodialysis patients [41,42].

In the present study, the survivor group showed significantly higher energy intake than the non-survivor group (26.6 ± 5.6 kcal/kg vs. 24.3 ± 4.2 kcal/kg; *p* = 0.018). This finding is consistent with the recommended lower limit of average energy intake of 25.7 kcal/kg by Kidney Disease Outcome Quality Initiative Nutrition Clinical Practice Guideline [9] and with two prior studies reporting that survivors had significantly higher energy intake (25.9–27.4 kcal/kg) than non-survivors (22.0–23.5 kcal/kg) in ESRD patients with maintenance hemodialysis [27,28].

In addition, the present study reported that the survivor group had significantly higher protein intake than the non-survivor group (0.91 ± 0.22 g/kg vs. 0.82 ± 0.24 g/kg; *p* = 0.038). However, the protein nutrient density (g protein/1000 kcal) was not significantly different between the two groups. The protein intake <0.8 g/kg was associated with all-cause mortality in univariate analysis but not in multivariable analysis after adjusting for age, sex, Kt/V, diabetic mellitus and CCI in the present study. Previous studies have consistently demonstrated that survivors consumed significantly higher protein than non-survivors (1.01 ± 0.38 g/kg vs. 0.92 ± 0.34 g/kg; *p* = 0.02) [28], and that low protein intake (or decreased protein intake over time) was associated with increased risk for death in maintenance hemodialysis patients [41]. Antunes et al. [27] reported that protein intake <1.2 g/kg was a predictor of mortality in dialysis patients, but protein intake <0.8 g·kg was not a predictor of mortality in hemodialysis patients in the present study. Inconsistent results between the studies could be partly due the cutoff of protein intake (1.2 g/kg vs. 0.8 g/kg) and type of dialysis (dialysis vs. hemodialysis). In addition, Lorenzo et al. [15] reported that under-nutrition of energy was the major abnormality of muscle wasting rather than protein under-nutrition in stable chronic hemodialysis patients.

Indirect measurements of protein intake and nPCR are significantly associated with decreased mortality [29,43]. However, nPCR can be affected by underestimation of the following variables: the permeability of the dialyzer; the amount of blood; the dialysate flow rate; and the distribution of urea in obese, malnourished, or edematous patients [44]. In contrast, the nPCR can be overestimated by subsequent urea rebound after dialysis [45]. In this study, the survivor group had significantly higher nPCR than did the non-survivor group (1.10 ± 0.24 g/kg vs. 1.01 ± 0.21 g/kg; *p* = 0.034); however, the nPCR was not a significant predictor of survival in Cox proportional analysis in this study population.

Malnutrition in ESRD patients on maintenance hemodialysis may be a result of inadequate dietary intake caused by anorexia, underlying illness, psychosocial conditions, aging or chronic inflammation [46,47].

Davies et al. [29] reported that comorbidities were more significant independent predictors of mortality in peritoneal dialysis patients than were dietary protein and energy intake. However, the comorbidities in the survivor group may be less severe (according to the CCI) than those in the non-survivor group, which could potentially influence dietary intake (4.18 ± 1.75 vs. 5.92 ± 2.06; *p* < 0.001)**.**

Anthropometric and body composition parameters are also known independent predictors of mortality in hemodialysis patients [48]. Although the MAMC was significantly higher in the male survivor group compared to that in the male non-survivor group, this trend did not hold true in the in the other groups. This discrepancy may be attributed to the fact that these anthropometry and body composition measurements were only performed once at the start of the study.

Serum albumin level was not an independent variable associated with all-cause mortality after adjusting for age, sex, Kt/V, and CCI in this study. Instead, serum albumin level was more likely to be a measurement of disease severity than of malnutrition [49]. The hypoalbuminemia that is often observed in ESRD patients on maintenance hemodialysis may be a response to the release of cytokines, caused by acute and/or chronic inflammation, rather than a consequence of malnutrition alone [50].

This study has several limitations that should be considered in interpretation of our findings. First, the sample size was small and included subjects from a single center. In addition, given that it is an observational study, we cannot account for unmeasured and residual confounding. A third limitation is that we used the BIA technique to measure body composition, while dual-energy X-ray absorptiometry is often considered the gold standard [51].

## 5. Conclusions

In conclusion, our data suggest that energy intake and MIS are associated with 10-year mortality in ESRD patients on maintenance hemodialysis. Therefore, energy intake should be monitored regularly in these patients.

## Figures and Tables

**Table 1:** Comparison of patient characteristics between the survivor and non-survivor groups.

Variable	Total (*n* = 144)	Survivor (*n* = 91)	Non-Survivor (*n* = 53)	*p*-Value
Male, *n* (%)	77 (53.5)	41 (45.1)	36 (67.9)	0.010
Age (years)	57.9 ± 12.5	53.5 ± 12.2	65.3 ± 9.1	<0.001
Cause of ESRD, *n* (%)				0.043
Hypertension	36 (25.0)	21 (23.1)	15 (28.3)	
Diabetes mellitus	67 (46.5)	36 (39.6)	31 (58.5)	
Glomerular nephritis	13 (9.1)	12 (13.2)	1 (1.9)	
Polycystic kidney disease	3 (2.1)	3 (3.3)	0 (0.0)	
Unknown	25 (17.4)	19 (20.9)	6 (11.3)	
Years of dialysis	4.26 ± 4.00	3.97 ± 3.88	4.83 ± 4.20	0.260
Kt/V	1.70 ± 0.39	1.75 ± 0.45	1.60 ± 0.23	0.041
Comorbidity, *n* (%)				
Coronary artery disease	28 (19.4)	11 (12.1)	17 (32.1)	0.006
Congestive heart failure	19 (13.2)	8 (8.8)	11 (20.8)	0.071
Peripheral vascular disease	2 (1.4)	1 (1.1)	1 (1.9)	0.602
Cerebral vascular disease	14 (9.7)	7 (7.9)	7 (13.5)	0.382
Chronic pulmonary disease	2 (1.4)	2 (2.2)	0 (0.0)	0.532
Connective tissue disorder	6 (4.2)	4 (4.4)	2 (3.8)	1.000
Peptic ulcer disease	20 (13.9)	9 (9.9)	11 (20.8)	0.083
Liver disease	12 (8.3)	6 (6.6)	6 (11.3)	0.725
Diabetes mellitus	74 (51.3)	42 (46.2)	32 (60.5)	0.051
Hemiplegia	2 (1.4)	1 (1.1)	1 (1.9)	1.000
Charlson comorbidity index	4.72 ± 2.07	4.06 ± 1.74	5.85 ± 2.11	<0.001
Cause of death				
Cardiovascular cause			3 (5.7)	
Sudden cardiac death			13 (24.5)	
Cerebrovascular disease			4 (7.5)	
Infection			13 (24.5)	
Malignancy			2 (3.8)	
Suicide or other			1 (1.9)	
Unknown			17 (32.1)	

Values are expressed as mean ± SD or number of participants (percentage). ESRD: end stage renal disease.

**Table 2:** Comparisons in the anthropometric measurements and body compositions in end-stage renal disease patients on maintenance hemodialysis according to sex.

Variable	Male				Female			
	Total (*n* = 77)	Survivor (*n* = 41)	Non-Survivor (*n* = 36)	*p*-Value	Total (*n* = 67)	Survivor (*n* = 50)	Non-Survivor (*n* = 17)	*p*-Value
BMI (kg/m^2^)	22.1 ± 2.6	22.3 ± 2.6	21.7 ± 2.6	0.329	21.1 ± 3.4	20.8 ± 3.3	21.9 ± 3.4	0.225
TSF (mm)	7.8 ± 3.5	7.8 ± 3.5	7.8 ± 3.4	0.916	12.3 ± 6.2	12.2 ± 6.2	12.4 ± 6.5	0.904
MAMC (cm)	26.3 ± 2.2	26.8 ± 2.1	25.7 ± 2.1	0.027	23.6 ± 1.9	23.5 ± 2.2	23.7 ± 1.3	0.759
PBF (%)	20.0 ± 7.5	18.7 ± 8.3	21.6 ± 6.1	0.090	26.6 ± 10.3	26.1 ± 9.9	28.2 ± 9.9	0.461
TBFI (kg/m^2^)	4.8 ± 1.9	4.8 ± 2.2	4.9 ± 1.6	0.852	5.9 ± 3.0	5.7 ± 2.9	6.3 ± 3.4	0.498
MMI (kg/m^2^)	9.5 ± 1.5	9.7 ± 1.8	9.2 ± 1.1	0.159	8.1 ± 0.9	8.1 ± 0.9	8.0 ± 1.0	0.705

Values are expressed as mean ± SD. TSF, triceps-skinfold thicknesses; MAMC, mid arm muscle circumference; PBF, percent body fat; TBFI, total body fat index; MMI, muscle mass index.

**Table 3:** Comparisons of malnutrition inflammation score (MIS) and laboratory variables in end-stage renal disease patients on maintenance hemodialysis.

Variable	Total (*n* = 144)	Survivor (*n* = 91)	Non-Survivor (*n* = 53)	*p*-Value
MIS	5.5 ± 2.3	5.2 ± 2.3	6.1 ± 2.1	0.039
nPCR (g/kg/day)	1.07 ± 0.23	1.10 ± 0.24	1.01 ± 0.21	0.048
Hemoglobin (g/dL)	10.8 ± 1.1	10.7 ± 1.0	10.9 ± 1.2	0.271
Albumin (g/dL)	3.47 ± 0.28	3.50 ± 0.28	3.41 ± 0.28	0.080
Prealbumin (mg/dL)	29.5 ± 5.5	30.1 ± 5.1	28.4 ± 5.9	0.083
Creatinine (mg/dL)	10.8 ± 2.9	11.2 ± 2.9	10.3 ± 2.6	0.057
Urea nitrogen (mg/dL)	72.7 ± 20.7	73.7 ± 21.3	71.1 ± 19.5	0.456
Calcium (mg/dL)	9.1 ± 0.6	9.2 ± 0.6	9.0 ± 0.5	0.042
Phosphorous (mg/dL)	4.91 ± 1.67	5.07 ± 1.80	4.64 ± 1.40	0.136
C-reactive protein (μg/dL)	0.27 ± 0.50	0.25 ± 0.50	0.31 ± 0.50	0.511
Zinc (μg/dL)	77.2 ± 14.7	76.6 ± 15.1	76.6 ± 14.2	0.689
Iron (μg/dL)	73.7 ± 37.4	74.1 ± 36.7	72.9 ± 38.9	0.863
TIBC (μg/dL)	189.4 ± 29.9	189.2 ± 26.2	189.7 ± 35.9	0.924
Ferritin (ng/mL)	635.1 ± 425.1	590.3 ± 415.4	711.2 ± 434.9	0.101

Values are expressed as mean ± SD. MIS, malnutiriton inflammation score; nPCR, normalized protein catabolic rate to body weight; TIBC, total iron binding capacity.

**Table 4:** Comparisons of nutritional intake in end-stage renal disease patients on maintenance hemodialysis.

Variable	Total (*n* = 144)	Survivor (*n* = 91)	Non-Survivor (*n* = 53)	*p*-Value
Energy (kcal/kg/day)	25.8 ± 5.4	26.7 ± 5.8	24.3 ± 4.2	0.009
Protein (g/kg/day)	0.88 ± 0.23	0.91 ± 0.21	0.82 ± 0.24	0.020
Protein (g/1000 kcal)	34.0 ± 5.8	34.2 ± 5.7	33.7 ± 6.0	0.593
Energy intake/energy requirement (%)	75.9 ± 15.8	77.5 ± 16.3	73.5 ± 14.6	0.116
Protein intake/protein requirement (%)	72.5 ± 20.6	75.3 ± 20.1	67.9 ± 20.7	0.037
Carbohydrate (%)	60.3 ± 7.0	60.8 ± 7.1	59.4 ± 6.5	0.237
Protein (%)	13.7 ± 2.4	13.7 ± 2.4	13.5 ± 2.4	0.452
Fat (%)	25.9 ± 5.1	25.4 ± 5.4	26.7 ± 4.2	0.127

Values are expressed as mean ± SD.

**Table 5:** Univariate Cox proportional hazards analysis of all-cause mortality in end-stage renal disease patients on maintenance hemodialysis.

Predictor Variable		Hazard Ratio (95% CI)	*p*-Value
Non-nutritional	Age	1.080 (1.048,1.112)	<0.001
	Male	1.977 (1.110,3.522)	0.021
	Years of dialysis	1.041 (0.976,1.110)	0.225
	Diabetes mellitus	1.814 (1.046,3.147)	0.034
	CCI	1.335 (1.203,1.481)	<0.001
	Kt/v	0.395 (0.160,0.974)	0.044
Nutritional	MIS (>5 vs. ≤5)	2.249 (1.289, 3.924)	0.004
	BMI (<23 vs. ≥23 kg/m^2^)	1.031 (0.579, 1.836)	0.917
	nPCR (<1.0 or >1.4. vs. 1–1.4 g/kg/day)	1.087 (0.634, 1.864)	0.760
	Albumin (<3.8 vs. ≥3.8 g/dL)	2.167 (0.782, 6.044)	0.137
	Energy intake (<25 vs. ≥25 kcal/kg)	2.314 (1.326, 4.038)	0.003
	Protein intake (<0.8 vs. ≥0.8 g/kg)	1.735 (1.012, 2.975)	0.045

CCI, Charlson’s comorbidity index; MIS, malnutrition inflammation score; Kt/V, urea clearance over time; nPCR, normalized protein catabolic rate to body weight; CI, confidence interval.

**Table 6:** Multivariable Cox proportional hazards analysis of all-cause mortality in end-stage renal disease patients on maintenance hemodialysis.

	Predictor Variable	Hazard Ratio (95% CI)	*p*-Value
Model 1	MIS > 5	2.146 (1.173, 3.928)	0.013
	Energy intake <25 kcal/kg	1.860 (1.018, 3.399)	0.044
Model 2	MIS > 5	2.290 (1.240, 4.229)	0.008
	Protein intake (<0.8 g/kg)	1.345 (0.769, 2.353)	0.299

*p*-value was adjusted for age, sex, Kt/V, diabetic mellitus and Charlson comorbidity index.
